# Dataset on full width at half maximum of residual stress measurement of electron beam welded high strength structural steels (S960QL and S960M) by X-ray diffraction method

**DOI:** 10.1016/j.dib.2021.107341

**Published:** 2021-09-04

**Authors:** Raghawendra P.S. Sisodia, Marcell Gáspár, Máté Sepsi, Valéria Mertinger

**Affiliations:** a Institute of Materials Science and Technology, University of Miskolc, Miskolc, 3515, Hungary; b Institute of Physical Metallurgy, Metal forming and Nanotechnology, University of Miskolc, Miskolc, 3515, Hungary

**Keywords:** Electron beam welding (EBW), Residual stress, Full width at half maximum (FWHM), microstructure, high strength structural steels (HSSS), X-ray diffraction methods (XRD)

## Abstract

In this paper, we presented the dataset values of full width at half maximum (FWHM) with errors at each point corresponding to the value of longitudinal and transverse residual stress along the three lines for 14 points measured in the EBW welded joints (S960QL and S960M) of the related article [1]. This dataset is used to plot figures and describes their correspondence points with the interrelation of the residual stress graphs (Fig. 4) of the article [1]. The shape of the diffracted peak can be characterised in a simple way by the FWHM, which is the width in degree at half the peak height after background extraction. The measured width consist of instrumental and metallurgical broadening. The variation or increase in FWHM is resulted from the crystalline lattice defect e.g. solute foreign atoms, dislocations and grain boundary. Conversely, if we can determine the physical broadening, we get more information about the structure of the investigated material. In addition, the optical microscopic image of the base materials and weld microstructure are the other parts of the data. Diffraction data were collected using centreless X-ray diffraction (XRD) during *in situ* residual stress measurement of high strength structural steels S960QL and S960M. A more detailed interpretation of the data presented in this article is provided in article [1]. The presented data are produced as part of the main work entitled “Comparative evaluation of residual stresses in vacuum electron beam welded high strength steel S960QL and S960M butt joints [1]”.

**Specifications Table**

**Table:** 

Subject	Mechanical Engineering
Specific subject area	Electron beam welding, Residual stress, High strength structural steel
Type of data	Table Image Graph
How data were acquired	X-ray diffraction, Stresstech XStress Robot centreless X Ray diffractometer. Optical microscope, microstructure images were taken by using an Axio Observer D1m (Zeiss) inverted microscope.
Data format	Raw
Parameters for data collection	-For XRD test, Standard Cr-Kα source with 30 kV tube voltage and 8 mA tube current used to measure the ferrite interference line of {211} plane series. The Young modulus (E)= 211 GPa and the Poisson's ratio (ν) with 0.3 were considered - For optical microscopic analysis, magnification, *M* = 200x, *M* = 500x
Description of data collection	- XRD data was collected using 1 mm collimator size in diameter. 7/7 tilting applied between Ψ: -45°and +45° at each measuring point. - Optical microscopy was performed on the sample sectioned through the weld in transverse direction and then specimens etched with Nital (2% HNO_3_) for 10 s.
Data source location	Institute of Materials Science & Technology; Institute of Physical Metallurgy, Metal forming and Nanotechnology. University of Miskolc Miskolc, Borsod Abaúj Zemplén, 3515 Hungary
Data accessibility	With the article
Related research article	R.P.S. Sisodia, M. Gáspár, M. Sepsi, V. Mertinger, Comparative evaluation of residual stresses in vacuum electron beam welded high strength steel S960QL and S960M butt joints, Vacuum. 184 (2021) 109931. https://doi.org/10.1016/j.vacuum.2020.109931.

**Value of the Data**

• The residual stresses of different orders are present together in the material, often regardless of the cause. While the first-order stress causes the Bragg angle shift, the third-order stresses increase the FWHM value. Therefore, when examining the residual stress, it is advisable to monitor the change of both parameters as well, because we can obtain additional information about the conditions of hardening.
• The data will benefit researchers, structural and welding engineers, modelling engineers exploring deformation mechanisms, residual stress determination with different welding processes and their interaction in HSS e.g., S960QL and S960M.
• These data can be used further to compare the FWHM results with same grade high strength steel welded joint with different welding processes or higher strength steel grades welded joint with gas metal arc welding (GMAW) process or other welding processes to analyse the value of FWHM which is increased by everything that results from the defect of the crystalline lattice and causes a third order residual stress.
• Data can be used to compare the residual stress measurements made by other measurement techniques e.g., neutron diffraction or deep hole drilling methods.
• The data can be used to validate and calibrate future numerical modelling of residual stress distribution and FWHM.

## 1 Data Description

**Fig. 1:**
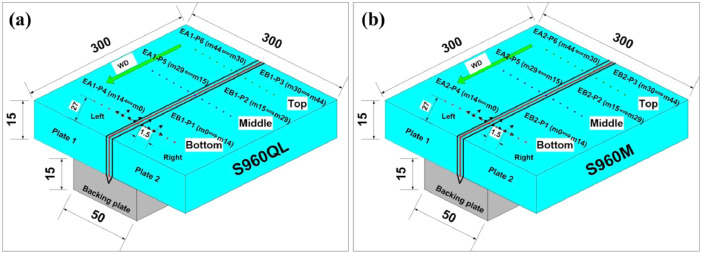
Schematic sketch of data point measurements for FWHM: (a) S960QL, and (b) S960M (WD= welding direction).

The data provided in this paper relate to the paper published in the Vacuum Journal [1]. The raw and analysed data on FWHM with scatter values at the corresponding points of the residual stresses measured at 14 different points each in three different lines of the welded joint shown in Fig. 3(b) [1] are presented in this article. The schematic diagram, Fig. 3(b) [1] provides the details scheme or pattern of the measured residual stress and their corresponding FWHM used for the X-ray diffraction method. The dataset used for plotting graphs in Fig. 4. are provided in.xls file (Raw data_1.xls & Raw data_2.xls) along with this article. The detail representation of the excel sheet data for BM S960QL (Raw data_1.xls) & BM S960M (Raw data_2.xls) are presented in schematic sketch Fig. 1a and b, respectively, for the convenience of the users. The different tab in excel sheet Raw data_1.xls (S960QL) which containing measurement data are represented by EA1-P6, EA1-P5, EA1-P4, EB1-P3, EB1-P2, EB1-P1 and m0 to m14, m15 to m29, m30 to m44 are the points of measurement which starts from weld toe to the edge of sample. Similarly, the different tab in excel sheet Raw data_2.xls (S960M) which containing measurement data are represented by EA2-P6, EA2-P5, EA2-P4, EB2-P3, EB2-P2, EB2-P1 and m0 to m14, m15 to m29, m30 to m44 are the points of measurement which starts from weld toe to the edge of sample on both side of the welded joint as shown in Fig. 1. Tables 2 & 3 represent the S960QL and S960M detail dataset of FWHM used for the graphs mentioned in Fig. 4 (a), (b) & Fig. 4(c) & (d), respectively.

## 2 Experimental Design, Materials and Methods

In this experiment, two base materials (BM) namely WELDOX 960 E (S960QL in EN 10025-6) and ALFORM 960M (S960M in EN 10149-2) with plate thickness 15 mm were used. Their chemical compositions and mechanical properties are presented in the article [1]. These two steels are differing in their carbon and micro-alloying element content and thus so in CEV and CET [2], [3], [4]. The plates with dimensions 300 × 150 × 15 mm (according to EN 15614-11:2002) in two pieces for butt welded joint were used under high vacuum of 2 × 10^−4^ mbar for electron beam welding using an EBOCAM EK74C–EG150-30BJ EBW machine. The electron beam welding process is an innovative and versatile technology [5]. The underlay plate with dimension of 300 × 50 mm is used for EBW process with not through penetration mode to obtain sound result, assembled with original butt welded joint. The edges of the samples and assembly unit of backing plate with butt joint plates were properly cleaned and milled to the maximum allowable gap of 0.15 mm. The electron beam welded joint without filler material with full penetration was obtained after several trials and the beam pattern was straight oscillation with the amplitude of 1 mm. The optimal welding parameters used in this investigation are same for the BM which are shown in Table 1.

**Table 1:** Optimal EBW welding parameters.

Steels	Accelerating voltage (kV)	Beam current (mA)	Welding speed (mm/s)	Beam diameter (mm)	Working distance (mm)	Linear heat input (kJ/mm)
S960QL/S960M	150	49	10	0.4	500	0.661

**Table 2:** Full width at half maximum (FWHM), S960QL, longitudinal & transverse.

Excel file name: Raw Data_1, FWHM														
EA1-P6 (Left)/Top line					EA1-P5 (Left)/Middle line					EA1-P4 (Left)/Bottom line				
	φ, 0.0, Long.		φ, -90.0, Trans.			φ, 0.0, Long.		φ, -90.0, Trans.			φ, 0.0, Long.		φ, -90.0, Trans.	
Point name	FWHM (°)	Scatter± (°)	FWHM (°)	Scatter± (°)	Point name	FWHM (°)	Scatter± (°)	FWHM (°)	Scatter± (°)	Point name	FWHM (°)	Scatter± (°)	FWHM (°)	Scatter± (°)
m44	3.09	0.30	3.00	0.30	m29	3.06	0.11	2.93	0.16	m14	2.97	0.15	2.94	0.13
m43	3.15	0.25	2.96	0.21	m28	3.09	0.17	2.98	0.10	m13	3.10	0.15	3.00	0.10
m42	3.07	0.32	2.87	0.23	m27	3.04	0.11	2.92	0.21	m12	2.94	0.11	2.80	0.11
m41	3.13	0.22	3.06	0.16	m26	3.19	0.11	3.14	0.13	m11	3.05	0.06	2.95	0.07
m40	3.09	0.41	3.01	0.19	m25	3.12	0.09	3.12	0.10	m10	2.97	0.09	2.87	0.12
m39	2.90	0.24	2.94	0.33	m24	3.03	0.07	3.02	0.11	m9	2.82	0.17	2.78	0.13
m38	3.16	0.28	3.00	0.19	m23	3.13	0.15	3.16	0.15	m8	2.92	0.12	2.85	0.09
m37	3.17	0.24	3.14	0.21	m22	3.15	0.15	3.15	0.11	m7	2.85	0.12	2.81	0.12
m36	3.18	0.18	3.24	0.10	m21	3.04	0.19	3.05	0.12	m6	2.96	0.10	2.91	0.08
m35	3.10	0.11	3.04	0.15	m20	3.08	0.16	3.07	0.16	m5	2.93	0.16	2.92	0.19
m34	3.23	0.08	3.08	0.10	m19	2.62	0.21	2.81	0.35	m4	2.81	0.08	2.81	0.08
m33	2.60	0.08	2.63	0.13	m18	2.50	0.07	2.50	0.06	m3	2.63	0.04	2.74	0.09
m32	2.64	0.05	2.66	0.06	m17	2.49	0.04	2.51	0.04	m2	2.70	0.06	2.83	0.05
m31	2.45	0.04	2.46	0.07	m16	2.42	0.04	2.47	0.04	m1	2.63	0.05	2.74	0.07
m30	2.29	0.05	2.38	0.10	m15	2.31	0.03	2.36	0.06	m0	2.56	0.02	2.65	0.06

Fusion zone														
EB1-P3 (Right)/Top line					EB1-P2 (Right)/Middle line					EB1-P1 (Right)/Bottom line				

m30	2.31	0.04	2.37	0.06	m15	2.23	0.05	2.24	0.08	m0	2.23	0.03	2.26	0.08
m31	2.41	0.04	2.42	0.04	m16	2.39	0.04	2.39	0.04	m1	2.40	0.02	2.42	0.05
m32	2.48	0.05	2.48	0.04	m17	2.42	0.04	2.43	0.05	m2	2.42	0.04	2.43	0.05
m33	2.49	0.04	2.49	0.04	m18	2.41	0.05	2.42	0.06	m3	2.43	0.08	2.46	0.05
m34	2.81	0.31	2.84	0.19	m19	2.54	0.11	2.60	0.16	m4	2.72	0.11	2.72	0.23
m35	3.08	0.28	3.03	0.22	m20	2.88	0.24	2.87	0.28	m5	3.20	0.13	3.13	0.06
m36	2.93	0.29	2.84	0.27	m21	3.06	0.29	3.00	0.26	m6	3.13	0.14	2.99	0.19
m37	3.07	0.22	2.96	0.30	m22	2.97	0.26	2.98	0.25	m7	3.21	0.16	3.25	0.14
m38	2.94	0.30	2.96	0.27	m23	3.04	0.24	3.09	0.26	m8	3.34	0.19	3.22	0.22
m39	2.94	0.27	2.90	0.28	m24	3.11	0.26	3.01	0.28	m9	3.16	0.18	3.16	0.14
m40	3.11	0.22	3.02	0.22	m25	3.06	0.22	3.01	0.29	m10	3.20	0.17	3.17	0.23
m41	3.22	0.18	3.11	0.21	m26	3.10	0.29	3.12	0.26	m11	3.18	0.20	3.16	0.13
m42	3.23	0.19	3.05	0.18	m27	3.04	0.34	3.07	0.29	m12	3.10	0.29	3.05	0.22
m43	3.04	0.17	3.08	0.27	m28	3.04	0.31	3.06	0.24	m13	3.17	0.16	3.09	0.16
m44	3.12	0.13	3.22	0.10	m29	3.09	0.31	3.01	0.24	m14	3.05	0.14	3.08	0.10

**Table 3:** Full width at half maximum (FWHM), S960M, longitudinal & transverse.

Excel file name: Raw Data_2, FWHM														
EA2-P6 (Left)/Top line					EA2-P5 (Left)/Middle line					EA2-P4 (Left)/Bottom line				
	φ, 0.0, Long.		φ, -90.0, Trans.			φ, 0.0, Long.		φ, -90.0, Trans.			φ, 0.0, Long.		φ, -90.0, Trans.	
Point name	FWHM (°)	Scatter± (°)	FWHM (°)	Scatter± (°)	Point name	FWHM (°)	Scatter± (°)	FWHM (°)	Scatter± (°)	Point name	FWHM (°)	Scatter± (°)	FWHM (°)	Scatter± (°)
m44	2.31	0.04	2.37	0.15	m29	2.28	0.02	2.32	0.04	m14	2.35	0.02	2.34	0.04
m43	2.26	0.04	2.27	0.04	m28	2.30	0.04	2.27	0.03	m13	2.37	0.03	2.34	0.04
m42	2.25	0.03	2.24	0.02	m27	2.24	0.04	2.26	0.04	m12	2.38	0.02	2.35	0.03
m41	2.24	0.03	2.23	0.04	m26	2.32	0.04	2.32	0.04	m11	2.32	0.04	2.31	0.04
m40	2.24	0.03	2.24	0.04	m25	2.24	0.03	2.28	0.04	m10	2.34	0.03	2.35	0.05
m39	2.26	0.04	2.25	0.03	m24	2.26	0.02	2.27	0.05	m9	2.31	0.02	2.32	0.03
m38	2.22	0.03	2.24	0.05	m23	2.30	0.03	2.31	0.04	m8	2.35	0.05	2.36	0.04
m37	2.19	0.03	2.21	0.02	m22	2.28	0.06	2.26	0.04	m7	2.33	0.04	2.31	0.04
m36	2.24	0.03	2.27	0.04	m21	2.33	0.02	2.33	0.04	m6	2.34	0.06	2.31	0.03
m35	2.44	0.06	2.44	0.10	m20	2.34	0.03	2.37	0.03	m5	2.31	0.03	2.32	0.04
m34	2.53	0.05	2.55	0.07	m19	2.46	0.03	2.50	0.04	m4	2.51	0.04	2.50	0.06
m33	2.56	0.06	2.57	0.07	m18	2.64	0.06	2.65	0.07	m3	2.66	0.06	2.66	0.06
m32	2.55	0.04	2.54	0.06	m17	2.59	0.04	2.63	0.06	m2	2.61	0.05	2.63	0.08
m31	2.44	0.04	2.46	0.07	m16	2.52	0.04	2.58	0.07	m1	2.55	0.05	2.60	0.06
m30	2.35	0.02	2.43	0.08	m15	2.43	0.03	2.50	0.07	m0	2.43	0.04	2.48	0.07

Fusion zone														

EB2-P3 (Right)/Top line					EB2-P2 (Right)/Middle line					EB2-P1 (Right)/Bottom line				

m30	2.29	0.03	2.36	0.07	m15	2.41	0.06	2.72	0.37	m0	2.59	0.04	2.72	0.17
m31	2.40	0.05	2.42	0.07	m16	2.50	0.03	2.54	0.06	m1	2.61	0.03	2.66	0.07
m32	2.48	0.07	2.48	0.06	m17	2.54	0.07	2.60	0.08	m2	2.70	0.05	2.72	0.08
m33	2.41	0.07	2.43	0.07	m18	2.60	0.07	2.63	0.09	m3	2.75	0.07	2.74	0.10
m34	2.15	0.05	2.19	0.08	m19	2.55	0.07	2.53	0.10	m4	2.70	0.10	2.71	0.11
m35	2.10	0.05	2.09	0.05	m20	2.50	0.07	2.44	0.10	m5	2.56	0.07	2.60	0.12
m36	2.06	0.04	2.05	0.03	m21	2.39	0.07	2.36	0.08	m6	2.61	0.11	2.56	0.10
m37	2.08	0.04	2.09	0.04	m22	2.37	0.10	2.38	0.07	m7	2.39	0.10	2.36	0.08
m38	2.08	0.04	2.09	0.04	m23	2.33	0.05	2.36	0.11	m8	2.26	0.05	2.22	0.04
m39	2.09	0.04	2.10	0.05	m24	2.29	0.07	2.29	0.06	m9	2.27	0.05	2.23	0.04
m40	2.08	0.03	2.06	0.03	m25	2.31	0.07	2.30	0.04	m10	2.27	0.06	2.26	0.03
m41	2.06	0.04	2.06	0.03	m26	2.39	0.07	2.40	0.07	m11	2.33	0.04	2.30	0.04
m42	2.06	0.03	2.04	0.03	m27	2.25	0.05	2.27	0.06	m12	2.41	0.08	2.38	0.08
m43	2.11	0.03	2.09	0.03	m28	2.34	0.11	2.27	0.04	m13	2.30	0.08	2.29	0.08
m44	2.05	0.02	2.05	0.03	m29	2.45	0.10	2.40	0.09	m14	2.34	0.07	2.30	0.08

**Fig. 2:**
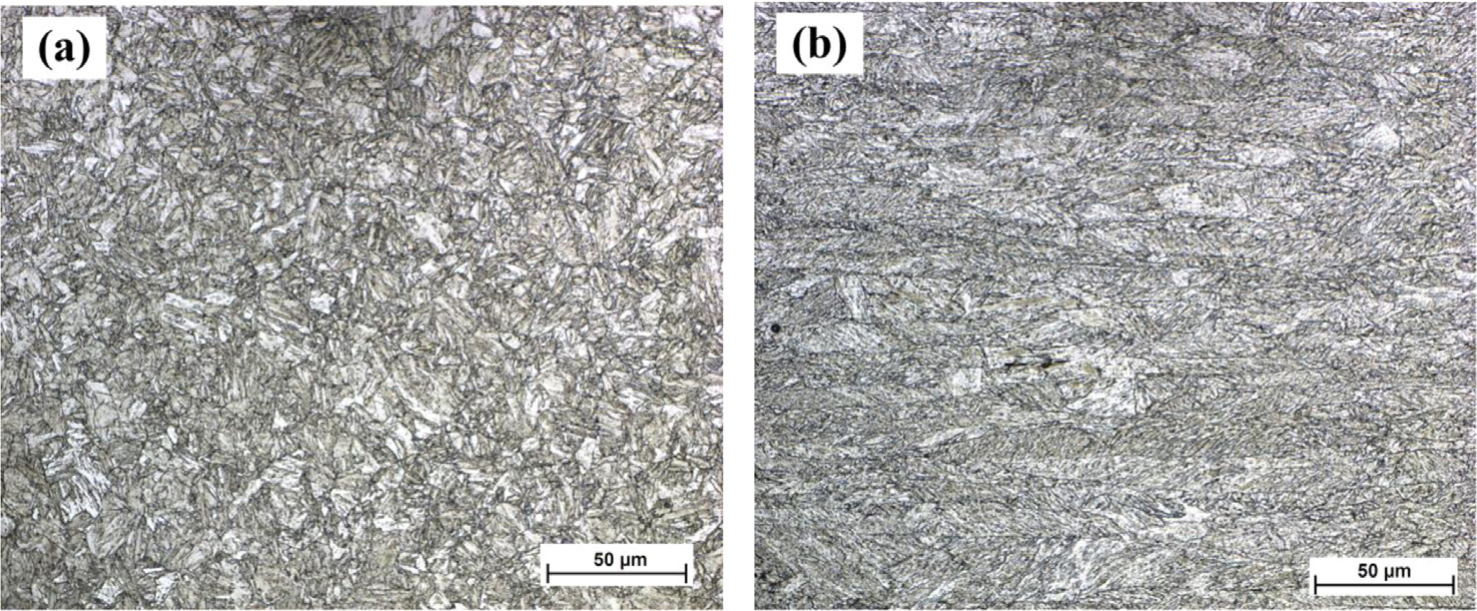
Microstructures of the base metals: (a) S960QL, and (b) S960M, *M* = 500x.

The metallographic samples for optical micrography observations were sectioned through the weld in transverse direction. The sectioned samples were polished with SiC waterproof papers in series of 120, 400, 800 & 2000 ANSI grit and finally with a disc using diamond paste of 1 µm. The specimens were then etched with Nital (2% HNO_3_) for 10 s to observe microstructure in base materials and weld materials. The resulting image of optical microstructures (*M* = 500x) of S960QL and S960M base material are shown in Fig. 2a and b, respectively. The microstructure of S960QL base material is consists of tempered martensite (TM) and bainite and of S960M consists of upper bainite (B_U_) and tempered martensite (TM) [6], shown in Fig. 2a & Fig. 2b, respectively.

**Fig. 3:**
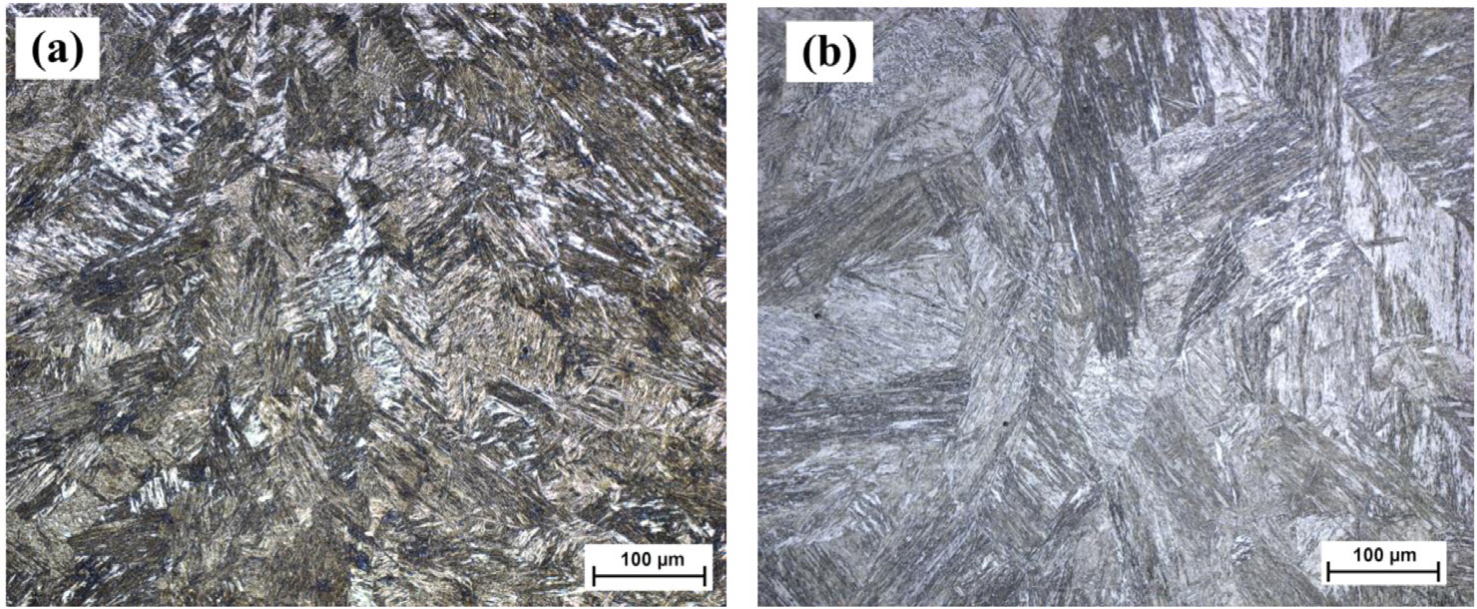
Optical micrographs of weld centre of EBW joints: (a) S960QL, and (b) S960M, *M* = 200x.

Optical micrographs of weld centre of the S960QL and S960M EBW joints are shown in Fig. 3a and b, respectively. The S960QL fusion zone reveals that it consists of fine dendritic martensite grains (Fig. 3a) which are perpendicular to the weld centre line (weld pool) while S960M shows mainly martensitic microstructure is clearly observed in Fig. 3(b).

**Fig. 4:**
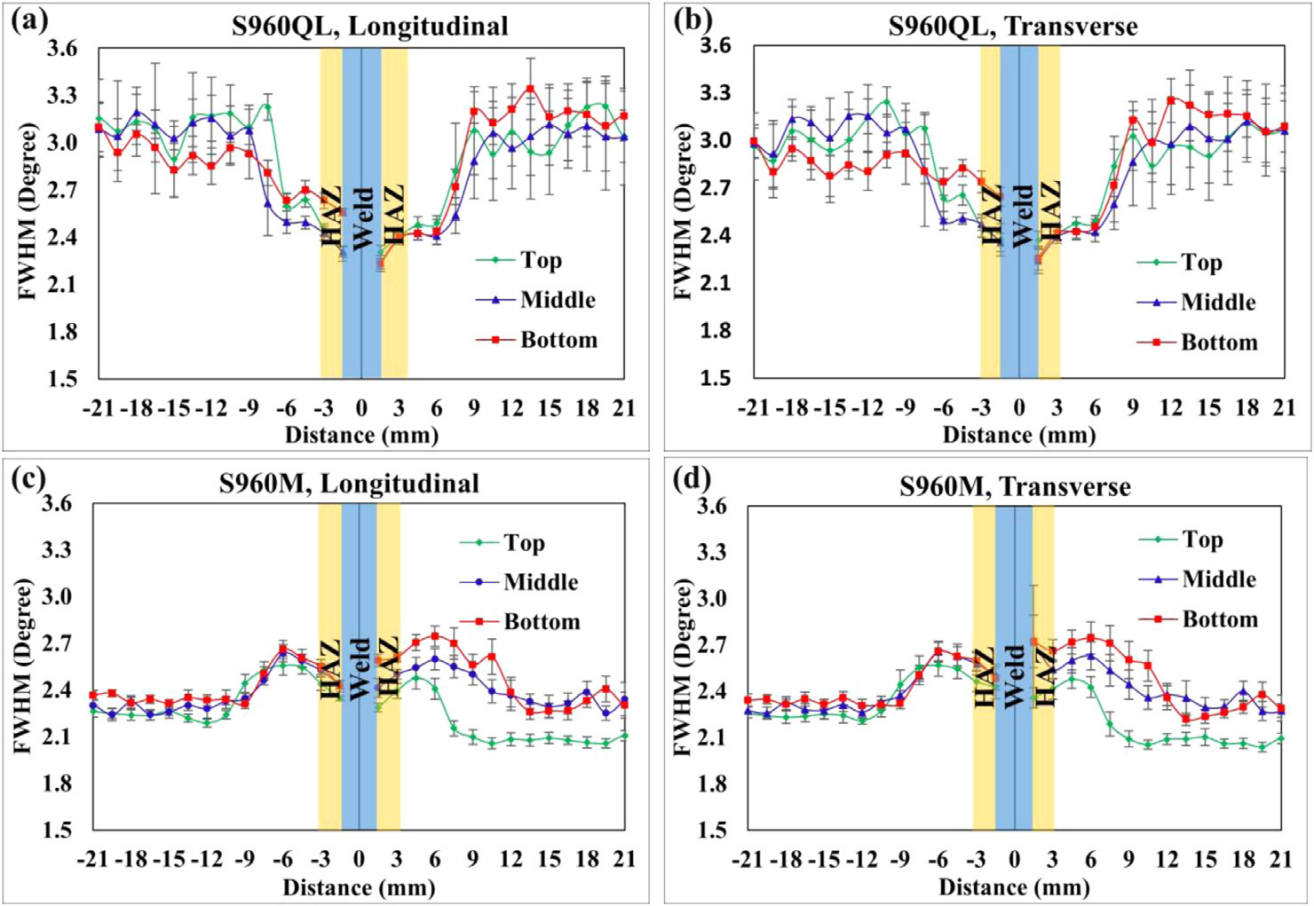
Full width at half depth: S960QL (a), Longitudinal and (b) Transverse; S960M (c), Longitudinal and (d) Transverse.

Residual stress measurements are particularly important for the introduction of advanced joining processes, such as electron beam welding, friction stir welding etc., into commercial usage [7]. In turn, it helps to understand FWHM profile variation of the as-received measurement along the measured points of the welded joint to correlate well with the residual stress corresponding result.

Then after, FWHM distribution for the corresponding points of residual stress (RS) were measured at 14 measurement points with distance of 1.5 mm between each point in three lines i.e., top, middle and bottom by the X-ray diffraction method on full size EB welded specimen in the as-welded state by Stresstech XStress Robot centreless X Ray diffractometer and the detailed schematic of the pattern of RS measurement and experimental procedure are provided in article [1].

This method of measurements is advantageous in carried out accordance to the well-known sin^2^ Ψ technique [8] (where Ψ is the angle between the diffracting planes and the specimen surface) where small depth of penetration signify that the sampled region assumed to be in plane stress [9]. XRD method is also called as non- destructive stress measurement technique.

The characteristic's features of high strength structural steels like S960QL and S960M are make it exceptional for the application in the field of engineering structure and highly loaded constructional components like heavy duty trucks, cranes, bridges, mobile cranes etc. [10,11].

## CRediT Author Statement

**Raghawendra Sisodia:** Methodology, Data curation, Writing – original draft, Visualization, Investigation; **Marcell Gáspár:** Conceptualization, Supervision, Writing – reviewing & editing; **Máté Sepsi:** Data curation, Investigation; **Valéria Mertinger:** Writing – review & editing.

## Declaration of Competing Interest

The authors declare that they have no known competing financial interests or personal relationships which have or could be perceived to have influenced the work reported in this article.

## Appendix Supplementary materials

Image, application 1

## References

[1] Sisodia R.P.S., Gáspár M., Sepsi M., Mertinger V. (2021). Comparative evaluation of residual stresses in vacuum electron beam welded high strength steel S960QL and S960M butt joints. Vacuum.

[2] Schaupp T., Schröepfer D., Kromm A., Kannengiesser T. (2014). Welding residual stress distribution of quenched and tempered and thermo-mechanically hot rolled high strength steels. Adv. Mater. Res..

[3] Lukács J. (2019). Fatigue crack propagation limit curves for high strength steels based on two-stage relationship. Eng. Fail. Anal..

[4] Lukács J., Dobosy Á. (2019). Matching effect on fatigue crack growth behaviour of high-strength steels GMA welded joints. Weld. World.

[5] Wȩglowski M.S., Błacha S., Phillips A. (2016). Electron beam welding - techniques and trends - review. Vacuum.

[6] Sisodia R., Gáspár M. (2021). Experimental assessment of microstructure and mechanical properties of electron beam welded S960M high strength structural steel. Manuf. Lett..

[7] Withers P.J., Bhadeshia H.K.D.H. (2001). Residual stress part 2 – nature and origins. Mater. Sci. Technol..

[8] Ramana P.V., Reddy G.M., Mohandas T., A.V.S.S.K.S. Gupta (2010). Microstructure and residual stress distribution of similar and dissimilar electron beam welds – maraging steel to medium alloy medium carbon steel. Mater. Des..

[9] Withers P.J., Bhadeshia H.K.D.H. (2001). Residual stress. part 1– measurement techniques. Mater. Sci. Technol..

[10] Májlinger K., Kalácska E., Spena P.Russo (2016). Gas metal arc welding of dissimilar AHSS sheets. Mater. Des..

[11] Maurer W., Ernst W., Rauch R., Kapl S., Pohl A., Krüssel T., Vallant R., Enzinger N. (2012). Electron beam welding of a TMCP steel with 700 MPa yield strength. Weld. World.

